# Iron and Manganese Peptonate

**Published:** 1897-12

**Authors:** 


### IRON AND MANGANESE PEPTONATE.

Of the specific effect of iron in relieving "blood poverty" there can be no doubt. It is one of the oldest of empirical remedies, and modern research has made its administration a scientific certainty. The preparations of iron in general use are numerous; a novelty among new preparations is the compound of iron and manganese introduced some years ago by Dr. A. Gude, of Leipsic. The value of this combination has been proved by ten years' experience, and the fact is attested by direct and veiled indorsements running through all medical records of the subject during the last few years. Gould's American Year-book of Medicine, 1896, quotes an indorsement (p. 456), and similar references can be found in other annuals and medical publications.

An important report was published recently by Prof. von Ramdohr (*N. Y. Med. Journal*, June 26, 1897), quoting details of nineteen cases of gynecological operations, at St. Mark's and the Post-Graduate Hospitals, where Gude's preparation, known as Pepto-Mangan, was employed with "highly beneficial" results as the "quickest acting tonic to counteract anemia." The author concludes "that (1) it is beneficial to immediately put a patient on whom an operation has been performed on the use of an easily assimilated iron preparation, and (2) pepto-mangan seems to be such a rational ideal pharmaceutical preparation."

Another recent clinical report is that of Dr Gellhorn (*Therap. Monatshefie*, May 1897), who speaks of sixty cases of anemic conditions treated with pepto-mangan, obtaining "all that can be rationally demanded" from this "most efficient and useful auxiliary to our therapeutic efforts."

Dr. A. H. Roler details five cases of anemic young women (*Chicago Medical Recorder*, June, 1897,) successfully treated with pepto-mangan.

Dr. Henry A. Johnson, in *Archive of Pediatrics*, August, 1897, describes three cases of anemia following diarrhœa (children of seven, thirteen and twenty-four months), in which half teaspoonful doses of pepto-mangan, following rhubarb and soda, effected prompt improvement.

Reports of similar kind are numerous, and indicate that this is an efficient, assimilable and desirable iron preparation.

